# Using capillary electrophoresis to identify Anopheline species in routine sampling sites

**DOI:** 10.1002/ece3.10782

**Published:** 2024-03-12

**Authors:** Estelle Chabanol, Ottavia Romoli, Stanislas Talaga, Yanouk Epelboin, Katy Heu, Ghislaine Prévot, Mathilde Gendrin

**Affiliations:** ^1^ Microbiota of Insect Vectors Group Institut Pasteur de la Guyane Cayenne French Guiana; ^2^ Tropical Biome and Immunophysiopathology Laboratory Université de Guyane Cayenne French Guiana; ^3^ École Doctorale 587 Université de Guyane Cayenne French Guiana; ^4^ CNRS, Inserm, CHU de Lille, Institut Pasteur de Lille, U1019 – UMR 9017 – CIIL – Center for Infection and Immunity of Lille Université de Lille Lille France; ^5^ Department of Insect Vectors, Institut Pasteur Université de Paris Paris France

**Keywords:** *Anopheles*, capillary electrophoresis, French Guiana, ITS2, length polymorphism, mosquito, *Nyssorhynchus*, species identification

## Abstract

In the *Anopheles* genus, various mosquito species are able to transmit the *Plasmodium* parasites responsible for malaria, while others are non‐vectors. In an effort to better understand the biology of *Anopheles* species and to quantify transmission risk in an area, the identification of mosquito species collected in the field is an essential but problematic task. Morphological identification requires expertise and cannot be checked after processing samples in a destructive treatment, while sequencing of numerous samples is costly. Here, we introduce a method of Species identification via Simple Observation Coupled with Capillary Electrophoresis Technology (SOCCET). This molecular technique of species identification is based on precise determination of ITS2 length combined with a simple visual observation, the colour of mosquito hindleg tip. DNA extracted from field‐collected *Anopheles* mosquitoes was amplified with universal *Anopheles* ITS2 primers and analysed with a capillary electrophoresis device, which precisely determines the size of the fragments. We defined windows of amplicon sizes combined with fifth hind tarsus colour, which allows discrimination of the major *Anopheles* species found in our collections. We validated our parameters via Sanger sequencing of ITS2 amplicons. Using the SOCCET method, we characterised the composition of *Anopheles* populations in five locations of French Guiana, where we detected a total of nine species. *Anopheles braziliensis* and *Anopheles darlingi* were detected in four locations each and represented 13 and 67% of our samples, respectively. The SOCCET method can be particularly useful when working with routine sampling sites with a moderate species diversity, that is, when the number of local species is too high to define species‐specific primers but low enough to avoid individual ITS2 sequencing. This tool will be of interest to evaluate local malaria transmission risk and this approach may be further implemented for other mosquito genera.

## Graphical abstract

We developed the SOCCET method, allowing Species identification via simple Observation Coupled with Capillary Electrophoresis Technology. We describe its development and use to discriminate between nine *Anopheles* species of our samples collected in French Guiana.

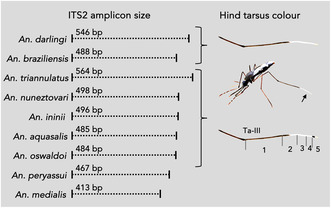

## 1 INTRODUCTION

Several species of mosquitoes are vectors of viruses or parasites causing serious diseases in humans. In particular, some species of *Anopheles* transmit the *Plasmodium* parasite, which causes malaria (Phillips et al., 2017) and thus strongly impacts human health with more than 247 million cases in 84 countries in 2021 (World Health Organization, 2022). Despite global efforts to reduce the burden of vector‐borne diseases, they remain a sanitary and economic threat in the intertropical area and beyond (World Health Organization, 2017). However, among the 3500 species of mosquitoes worldwide, only a minority are vectors of pathogens, therefore species identification is a critical element of surveillance studies.

The *Anopheles* genus, present on all continents, is subdivided into seven subgenera containing more than 450 species. Some of these species belong to complexes in which species boundaries are not always strict. The *Nyssorhynchus* subgenus, sometimes also considered as a genus, is distributed throughout tropical America and currently includes 40 formally described species (Strickman et al., 2021). In French Guiana, there are 245 known mosquito species (Talaga et al., 2020; Talaga & Gendrin, 2022) including 22 species of *Anopheles* (Talaga et al., 2015), nine of which belong to the *Nyssorhynchus* subgenus. Among them, *Anopheles darlingi* is considered the main malaria vector in South America (Hiwat & Bretas, 2011; Sinka et al., 2012) and *Anopheles aquasalis*, although not being incriminated as a vector in French Guiana, is also considered one of the principal vectors in neighbouring countries (Pimenta et al., 2015; Sinka et al., 2012). Moreover, *Anopheles medialis*, *Anopheles nuneztovari* and *Anopheles oswaldoi* have been found naturally infected with *Plasmodium* in French Guiana (Dusfour, Issaly, et al., 2012), as well as *Anopheles braziliensis* and *Anopheles triannulatus* in Brazil (Daniel‐Ribeiro et al., 1990; Duarte et al., 2013), but the real extent of their involvement in parasite transmission between humans is currently unknown. Given the known differences in their ability to transmit malaria, it is important to identify the exact species of *Anopheles* mosquitoes when studying and monitoring field population distributions and dynamics. Moreover, given the unclear role of some species as vectors, it is important to pursue sampling efforts, which may be combined with vector competence assays to develop a better knowledge of the role of diverse species assemblages in transmission cycles.

Originally, mosquito species identification was only based on visual observation of morphological characteristics with the help of dichotomous taxonomic keys (Coetzee, 2020; Gunathilaka, 2017; Sallum et al., 2020). This method is effective and relatively accessible when morphological differences are substantial, yet it is arduous and requires advanced skills when variations are subtle. The use of additional equipment and the dissection of internal structures are also sometimes necessary to distinguish between morphologically close species, as for *Culex* mosquitoes, in which meticulous dissection of male genitalia is required (de Sá et al., 2020; Sallum & Forattini, 1996). Moreover, even with properly trained and experienced professionals the task frequently remains a challenge, misidentifications are common (Rahola et al., 2022) and results cannot be double checked in case of doubt once samples have been processed in a destructive treatment. Finally, it requires physical manipulations that are not always compatible with experiments requiring fast sampling of specimens on the field, for instance, for OMIC analyses.

Alternative methods have been developed based on molecular biology techniques. *CO1* (Cytochrome C Oxidase Subunit 1), a mitochondrial gene, and ITS2 (Internal Transcribed Spacer 2), a non‐coding nuclear sequence located between *5.8S* and *28S* ribosomal RNA genes, are often used for molecular identification of animal species, as their species‐specific sequences are available in databases. Amplification of DNA regions with species‐specific sizes via multiplex PCR (Polymerase Chain Reaction) is often used as a substitute for sequencing to discriminate between a limited number of species found locally (Bang, Kim, et al., 2021; Bang, Won, et al., 2021; Brosseau et al., 2019; Wilai et al., 2020), notably *Anopheles coluzzii* and *Anopheles gambiae* found in Africa (Niang et al., 2015; Santolamazza et al., 2008) and other species from the *An. gambiae* complex (Scott et al., 1993; Wilkins et al., 2006). When a higher number of species are involved and the design of species‐specific primers is not feasible, PCR may be combined with additional treatment with restriction enzymes in the RFLP (Restriction Fragment Length Polymorphism) technique (Fanello et al., 2002; Vezenegho et al., 2022).

Other approaches for mosquito species identification are currently being considered. Mosquito protein profiling using MALDI‐TOF technology is promising (Sánchez‐Juanes et al., 2022), even though it requires a substantial investment in terms of equipment and databases are still under development (Costa et al., 2023). Nowadays, increasingly powerful software solutions make it possible to perform morphometry of mosquito wings (Lorenz et al., 2017) and the use of artificial intelligence may allow for automatic visual identification of mosquitoes (Goodwin et al., 2021). The sound and frequency of wing beats is also used to develop identification tools at the genus level, meant to be accessible on a simple smartphone (Mukundarajan et al., 2017). Yet the implementation of these tools needs more development and advanced computational knowledge.

So far, species identification via capillary electrophoresis, which finely detects size polymorphisms, has been developed to identify bacteria and yeasts or mammals, birds and fish in forensic studies (Gray et al., 2014; Nakamura et al., 2009; Pun et al., 2009; Turenne et al., 1999; Wehrhahn et al., 2012). As far as we know, this technology has never been used for insect identification before. In this study, we deployed this method to distinguish between *Anopheles* species from our routine sampling sites in French Guiana. The method is based on precise discrimination of Anophelines' natural ITS2 sequence size polymorphism using capillary electrophoresis, combined with a simple morphological observation, the colour of the hindleg tip (fifth hind tarsus, Ta‐III_5_). We introduce the SOCCET method, Species identification via simple Observation Coupled with Capillary Electrophoresis Technology. SOCCET is pronounced ‘socket’, similar to the French word ‘socquette’ (little sock), alluding to the fifth tarsus, yet it may be deployed for other species involving other morphological traits. This method limits the necessity for sequencing when mosquitoes are frequently sampled in known places with medium diversity.

## 2 MATERIALS AND METHODS

### 2.1 Mosquito collection

**FIGURE 1:**
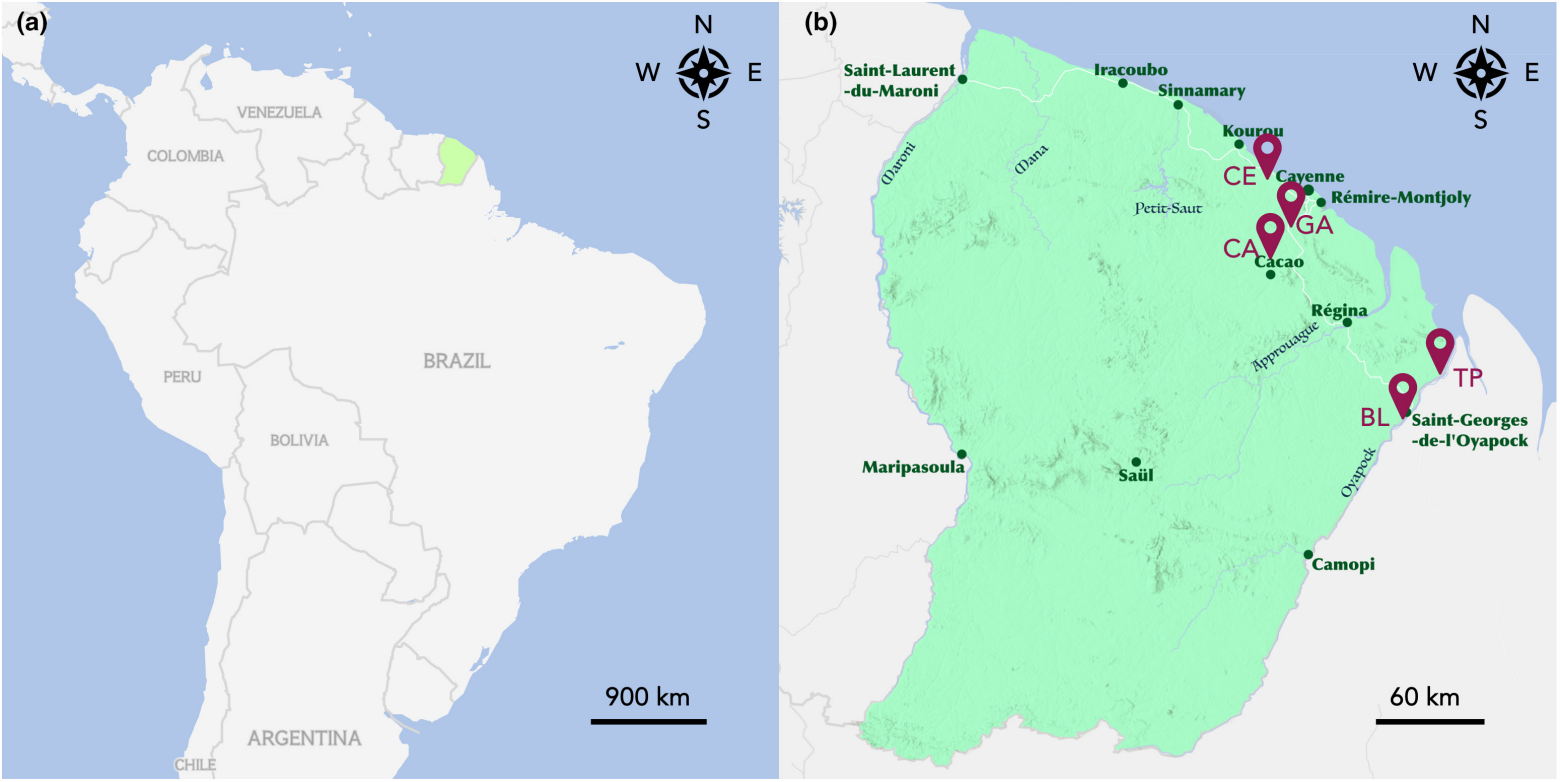
Maps of the geographical situation of French Guiana in South America (a) and mosquito sampling sites in French Guiana (b). La Césarée (CE), Le Galion (GA), Cacao (CA), Blondin (BL) and Trois Palétuviers (TP) are the five sampling locations. Maps are adapted from https://mapmaker.nationalgeographic.org. Copyright: Esri, HERE, Garmin, FAO, NOAA, USGS.

Mosquitoes were sampled in five different locations in French Guiana: La Césarée (CE, 5°0′57.32″ N, 52°31′41.36″ W); Le Galion (GA, 4°47′0.08″ N, 52°24′42.74″ W); Cacao (CA, 4°34′32.59″ N, 52°28′4.81″ W); Blondin (BL, 3°52′37.21″ N, 51°48′48″ W) and Trois Palétuviers (TP, 4°2′59.46″ N, 51°40′9.77″ W) (Figure 1). While CE, GA and CA are considered malaria‐free areas, BL and TP are located in a transborder region with higher risks of malaria resurgence. In 2017, a malaria outbreak was observed among inhabitants of TP village (Mosnier et al., 2020), thus it remains a region of interest with respect to malaria control programs.

Field missions took place over a 4‐year period, from June to November of 2018, 2019, 2020 and 2021. In French Guiana, these months correspond to the wet‐dry season transition and to the dry season, that is, when *Anopheles* densities are higher in the Amazonian region (Adde et al., 2017; Barbosa et al., 2014; Jules et al., 2019).

Mosquitoes were collected using Mosquito Magnet® traps (WoodStream™). These devices principally attract blood‐seeking female mosquitoes by mimicking host stimuli such as heat, CO_2_ and odour molecules (used here: octenol). The mosquitoes that come close enough are then trapped by an airflow into a net. Because *Anopheles* mosquitoes are nocturnal, traps were in operation from early evening to early morning, mainly during two sessions, from 5 to 10 p.m. and from 10 p.m. to 8 a.m., after each of which nets were changed and mosquitoes were recovered.

### 2.2 Mosquito visual inspection before dissection

All collected mosquitoes were transferred from trap nets into cages, where they were sorted out. Anophelinae were selected on the basis of their general appearance and behaviour: in contrast to mosquitoes from the Culicinae subfamily, their maxillary palps are about as long as their proboscis, they have slender bodies and, finally, most stand with the body inclined at an angle of 30–45° to the surface. Culicinae were discarded.

*Anopheles* were not identified visually at the species level, but the colour of their hind tarsi was checked, either by eye or under a stereomicroscope, and noted. Unfortunately, these data were not consistently collected during the first missions. We implemented its systematic collection since the last missions of 2019.

### 2.3 DNA extraction

After dissection, mosquito tissues were preserved in 70%–100% ethanol and kept at −80°C upon arrival at the laboratory and until further processing. DNA was extracted either from midguts with ZymoBiomics 96 MagBead DNA Kit (Zymo Research), which allows further analyses of microbiota composition or from carcasses and legs with HighPrep Blood & Tissue DNA Kit (MagBio), which is cheaper. Samples were mixed using 0.5 mm glass beads in 2 mL screwcap tubes with a Precellys Evolution (Bertin Technologies) bead beater homogeniser. Automatic DNA extraction was performed with the KingFisher Duo Prime system (Thermo Scientific). DNA was eluted in ZymoBiomics DNAse/RNAse Free Water from the kit for midguts and MB Elution Buffer was supplied in the kit for carcasses and legs. Extracted DNA was kept at −80°C until further utilisation.

### 2.4 ITS2 PCR amplification

PCR amplification of the ITS2 region was performed with Hot FirePol DNA Polymerase Kit (Solis Biodyne). Total PCR mix volume per sample was 50 μL with 48 μL premix and 2 μL DNA. The premix contained 36.8 μL H_2_O, 5 μL 10× Buffer B1, 3 μL 25 mM MgCl2, 1 μL 10 mM Deoxynucleotide (dNTP) Solution Mix (New England BioLabs), 1 μL 10 nM Forward ITS2 Primer, 1 μL 10 nM Reverse ITS2 Primer and 0.2 μL (5 U/μL) Hot FirePol DNA Polymerase. The DNA was either undiluted for extracted midguts and legs or diluted at 1/10 in DNAse‐/RNAse‐free water for extracted carcasses. *Anopheles* ITS2 primer sequences are 5′‐TGTGAACTGCAGGACACAT‐3′ (Forward) and 5′‐TATGCTTAAATTCAGGGGGTAG‐3′ (Reverse) (Saul & Beebe, 1995; Vezenegho et al., 2022). PCR was performed on a SimpliAmp Thermal Cycler (Applied Biosystems) using the cycle: 95°C × 15 min, (95°C × 30 s, 56°C × 45 s, 72°C × 40 s) × 35 cycles, 72°C × 10 min, 4°C × ∞. Amplified DNA was either used directly after or stored at 4°C or −20°C until use.

### 2.5 Agarose gel

1% agarose gel was made with 1 g UltraPure Agarose (Invitrogen), 100 mL 0.5× TBE Buffer diluted from TBE Buffer 10× (Biosolve) and 10 μL Midori Green Advance (Nippon Genetics). 8 μL of each sample was mixed with 2 μL DNA Gel Loading Dye 6× (Thermo Scientific) and 10 μL of 100 bp DNA Ladder Ready to Load size marker (Solis Biodyne) was used as reference. Migration took 45 min at 120 V with Owl EC300XL Compact Power Supply (Thermo Scientific). Gel visualisation and analysis were performed using Alliance Q9 Advanced imaging system and software (Uvitec Cambridge).

### 2.6 Capillary electrophoresis

Capillary electrophoresis was carried out in a QIAxcel Advanced System (Qiagen). QIAxcel DNA High Resolution Kit was chosen for higher precision and samples were run using OM800 method, QX Alignment Marker 15 bp–1 kb and QX DNA Size Marker 50–800 bp. Results were treated with QIAxcel ScreenGel Software and manually checked to ensure that detected peaks were real and that noise signals were eliminated. Reports were generated and ITS2 amplicon lengths were recovered for further analysis.

### 2.7 Sequencing and alignment

PCR products were kept in PCR plates sealed with adhesive film or caps and sent overseas for sequencing via fast shipping (Chronopost). Sanger sequencing was handled by Microsynth facility in Lyon, France. Forward and reverse sequencing were performed with the same set of primers as the PCR reaction. Fragment sequences were aligned using BLAST (NCBI, NIH) against the standard databases (nucleotide collection nr/nt) (Altschul et al., 1990; Sayers et al., 2022) and *Anopheles* species identifications were retrieved for forward and reverse sequences individually and integrated into our data and metadata files (Data S1).

Consensus sequences were generated using Geneious Prime® (2023.1.2 – Restricted) from forward and reverse sequences for 42 samples: 4 *An. aquasalis*, 8 *An. braziliensis*, 7 *An. darlingi*, 2 *An. ininii*, 6 *An. medialis*, 4 *An. nuneztovari*, 3 *An. oswaldoi*, 1 *An. peryassui* and 7 *An. triannulatus*. The sequences are accessible in GenBank (NCBI, NIH) database with accession numbers from OR149213 to OR149254 (Chabanol et al., 2023). Consensus sequences were aligned against sequences retrieved from the NCBI Nucleotide database (listed in Table S1) using Clustal Omega (https://www.ebi.ac.uk/Tools/msa/clustalo/; Data S2).

### 2.8 Analyses and graphics

Method development and routine analysis were mainly done in Excel tables (Data S3). The formula for species identification using capillary electrophoresis can be found in ‘Data S3’, supplementary file, column H. Figures were generated with R (version 4.2.1) and R Studio (version 2022.02.3 + 492) using Tidyverse (version 2.0.0) packages (Wickham et al., 2019), notably ggplot2 (version 3.4.2) package (Data S4 and S5).

Results shown in Table S3 were analysed by comparing the interval size of each triplicate on day 1 and on day 2 via the Wilcoxon rank sum test and Kruskal–Wallis rank sum test. Differences between variances were assessed via Levene's test centred on the median. In Figure S2, absolute values of the deviations of ITS2 lengths from the median were analysed via a Dunn (1964) Kruskal–Wallis multiple comparison, where *p*‐values are adjusted with the Holm method. These statistical analyses were performed using R Studio.

In the text, numbers are rounded to two significant figures.

## 3 RESULTS

Some of our field studies aim at collecting mosquitoes that can be used for metagenomic, metabolomic or transcriptomic analyses. In this context, we need to discriminate between *Anopheles* species that we sample in French Guiana, while allowing fast sampling. While we initially sequenced the ITS2 region of our specimens, we then considered taking benefit of natural differences in length of ITS2 sequence detected between *Anopheles* species in French Guiana. When analysing previously published ITS2 sequences of local *Anopheles* species (Gómez et al., 2015; Marrelli et al., 1999, 2005; Rosero et al., 2012; Sallum et al., 2008; Vezenegho et al., 2022), we observed that the amplicon length between the reference ITS2 primers ranges from 400 bp for *Anopheles eiseni* to 680 bp for *Anopheles minor* (Table S1).

**FIGURE 2:**
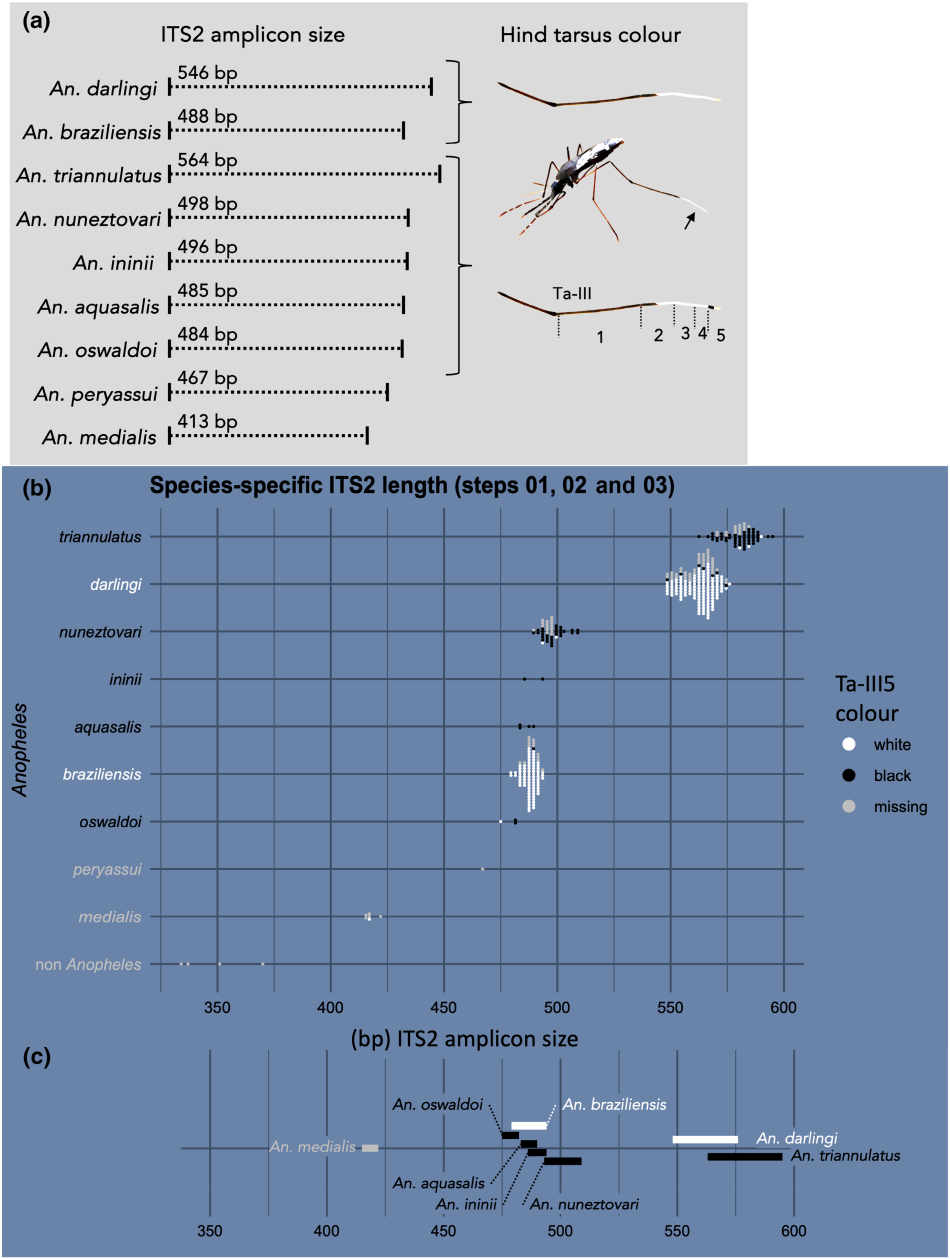
Definition of reference ITS2 amplicon lengths for the nine *Anopheles* species sampled in this study. (a) Reported ITS2 sequence size (from our consensus sequences, six of which matched reference [Vezenegho et al., 2022]) and tarsus colour of the hind leg of our species of interest. (b) Measured individual mosquito ITS2 amplicon lengths resulting from capillary electrophoresis analysis. Each dot represents a mosquito, and the dot colour matches the fifth hind tarsus colour information recorded at time of capture (white dots—fully white tarsus; black dots—including a black tarsus segment; some observations are erroneous) or missing (grey dots). (c) Size intervals defined for eight *Anopheles* species. Species were determined by sequencing during three development steps, allowing to link *Anopheles* species and ITS2 size.

An ITS2 sequence length is related to a unique species for *An. medialis* (413 bp), *Anopheles costai* (421 bp), *Anopheles nimbus* (442 bp), *Anopheles neivai* (477 bp), *Anopheles ininii* (495 bp), *An. nuneztovari* (498–500 bp), *An. triannulatus* (534, 536 or between 553 and 565 bp), *An. darlingi* (543–546 bp) and *An. minor* (680 bp). Some species, however, share similar ITS2 sequence lengths: *An. eiseni* (400–467 bp) with *Anopheles peryassui* (466–468 bp), *An. oswaldoi* (482–492 bp) with *An. aquasalis* (483–485 bp) and with *Anopheles marajoara* (487 bp) and *An. braziliensis* (487–488 bp). Hence, when some co‐occurring species share identical or similar ITS2 amplicon size, the species identification would be impossible using exclusively amplicon size information. We thus decided to additionally collect a simple morphological observation upon mosquito sampling in the field: the colour of their last three hind tarsi (Ta‐III_3,4,5_), in particular the fifth hind tarsus (Ta‐III_5_, at the tip of the hindleg) (Figure 2a). This morphological data do not require advanced skills in taxonomy but is enough to discriminate between some species with similar ITS2 lengths. *Anopheles braziliensis* and *An. darlingi* have their last three hind tarsi totally white while the other species like *An. aquasalis* and *An. oswaldoi* have a dark basal band on their fifth hind tarsus. Additionally, *Anopheles* outside of the *Nyssorhynchus* subgenus have a mix of white and black on their last three hind tarsi.

While differences in amplicon size are visible after PCR amplification and migration on an agarose gel, the analysis of gel images remains approximative and does not allow precise determination of fragment sizes (Figure S1A). With an agarose gel, fragment sizes are determined using the size marker as a reference, yet gel homogeneity and migration speed are not strictly controlled and slight differences in migration between wells cannot be corrected in the absence of internal controls. Capillary electrophoresis migration is another technique that allows automatization of the process and standardisation of the fragment size detection, using internal controls of defined sizes as references (Figure S1B). The precision is up to 3 bp for the most precise migration settings on fragments shorter than 500 bp. Additionally, the electropherogram allows verification of the quality of the fragment amplification (Figure S1C). We thus set up the SOCCET method to identify our *Anopheles* samples based on capillary electrophoresis analysis combined with observations of the fifth hind tarsus colour.

We collected *Anopheles* mosquitoes from five different locations in French Guiana (Figure 1) over a 4‐year period from 2018 to 2021. The colour of the fifth hind tarsus was noted for most mosquito samples at the time of capture, except for a third of the samples collected in 2019. Back in the laboratory, we extracted the DNA of each individual mosquito, amplified the ITS2 sequence and ran capillary electrophoresis.

Our method development was divided into three phases based on different mosquito collections from 2019 to 2021, with a first phase to define intervals (step 1; 2019) and two phases to adjust them (steps 2 and 3; 2020 and 2021 respectively) (Table 1). During step 1, we sequenced ITS2 in 167 *Anopheles* samples, identified species by BLAST and ran capillary electrophoresis to link mosquito species with observed ITS2 amplicon size. During steps 2 and 3, we collected 163 and 73 mosquitoes, respectively, tested the SOCCET process and verified the results by re‐sequencing 100% of our ITS2 amplicons. This enabled us to identify errors, adjust size intervals and add new species to the method. Finally, we applied this method routinely in our laboratory on a total of 372 samples (Table 1), consisting of a mix of samples collected from 2018 to 2021 that had not previously been identified. More than 99% could be identified without the need for sequencing. Only 2/372 samples needed to be sequenced because the SOCCET method did not conclusively identify the species.

**TABLE 1:** Methodological set‐up.

		*n*	Identification by the method	Identification by sequencing	Method analysis	Sequencing analysis	Verification by re‐sequencing
Method Development	Step 1	167	No (no intervals defined)	Yes	Definition of species‐specific intervals	Detection of problems or incoherences in sequencing results	19
	Step 2	163	Yes (with intervals defined at step 1)	Yes	Adjustment of intervals	Detection of problems or incoherences in sequencing results	21
	Step 3	73	Yes (with intervals adjusted at step 2)	Yes	Adjustment of intervals	Detection of problems or incoherences in sequencing results	1
Routine		372	Yes (with definitive intervals adjusted at step 3)	For uncertain results (2/372)	If necessary sequencing of 5% of samples for quality control		

*Note*: Sequencing of samples during method development allowed definition (step 1) and adjustment (steps 2 and 3) of species‐specific size intervals. In routine, sequencing only applies for uncertain species identification or quality control. Information about leg colour and ITS2 fragment size enabled us to detect discrepancies with sequencing results and the corresponding samples were sequenced again.

The ITS2 signal detected after capillary electrophoresis generally consisted of a single and well‐distinct peak on the electropherogram with the exception of *An. nuneztovari* mosquitoes for which approximately 80% of samples (37/46) had a profile consisting of multiple peaks, including a main peak around 497 bp. This pattern appeared specific to *An. nuneztovari* and may be explained by the propensity of some *An. nuneztovari* mosquitoes have intragenomic heterogeneity on their multiple copies of the ITS2 region, leading to length polymorphism within individuals (Onyabe & Conn, 1999). Additionally, four non‐*Anopheles* mosquito samples were mistakenly included within the first sample set (three *Psorophora* and one *Culex*) and gave rise to multiple‐peak profiles around 350 bp (Figure 2b), which were clearly distinguishable from any *Anopheles* mosquitoes and easily detected as unknown species by the method.

Each ITS2 size interval was set at step 1 from the minimum to the maximum size detected among all the samples of a species. Six *Anopheles* species were detected among the 167 samples of step 1, namely *An. braziliensis*, *An. darlingi*, *An. ininii*, *An. medialis*, *An. nuneztovari* and *An. triannulatus* (Figure 2b; Table S2). *Anopheles aquasalis* and *An. peryassui* were added at step 2 and *An. oswaldoi* at step 3 (Figure 2b; Table S2). Some species intervals were also slightly modified at steps 2 and 3 according to new data sets, with 0‐to‐6‐bp adjustments at either side of the intervals (Table S2). At the end, final amplicon size intervals were defined for eight *Anopheles* species and a single observation was collected for *An. peryassui* (Figure 2c).

In Figure 2b, the capillary electrophoresis migration appears more accurate for shorter fragments compared to longer sequences. We tested this observation, focusing on the four species with a high number of specimens, namely *An. braziliensis*, *An. darlingi*, *An. nuneztovari* and *An. triannulatus*. A Levene's test centred on medians showed that variances were significantly different between species (*p* < .001, *F* = 10.1). When comparing differences between observed sizes and respective medians, we observed that these differences were smaller for *An. braziliensis* (observed length median: 488 bp) and *An. nuneztovari* (497 bp) than for *An. darlingi* (564 bp) and *An. triannulatus* (581 bp) (Figure S2). Such a reduction in precision of the capillary electrophoresis when fragments are longer than 500 bp is consistent with indications of the manufacturer.

We checked that intervals were explained by imprecisions of capillary electrophoresis rather than by sequence variations within each species. Firstly, we built consensus sequences of forward and reverse sequences for 42 samples spanning all nine species (Chabanol et al., 2023). We saw no or negligible difference in size between samples taken from the beginning, middle and end of their species intervals. Secondly, we amplified and ran again *An. darlingi* and *An. triannulatus* DNA samples from both extremes of their respective intervals. We found no significant difference in size when these samples were processed in the same PCR and run in the same capillary electrophoresis batch (Table S3). An overnight −20°C storage increased interval amplitude by 4–5 bp (Table S3—*p* = .0023—*W* = 47.5, Wilcoxon rank sum test). Hence, we suspect that variations may be partly explained by the effect on DNA integrity of sample storage at 4°C or −20°C for one or several days before migration. Consistently with previously published data, freezing these DNA samples once overnight did not result in reduced band size, but we expect that multiple freeze–thaw cycles would do so (Shao et al., 2012).

Some overlaps appeared between the size intervals of several *Anopheles* species (Figure 2c). The intervals of *An. darlingi* and *An. triannulatus* overlapped between 563 and 576 bp, but the leg colour distinction made it possible to discriminate between these species. Similarly, the intervals of *An. braziliensis* and *An. oswaldoi* overlapped between 479 and 482 bp, but again the leg colour distinction allowed species identification. Several species‐specific intervals overlapped between 483 and 494 bp, for *An. aquasalis*, *An. braziliensis*, *An. ininii* and *An. nuneztovari*. *Anopheles braziliensis* can be distinguished from the rest by their fifth hind tarsus colour and *An. nuneztovari* mosquitoes by their tendency to generate multiple peak profiles. For the case of *An. aquasalis* and *An. ininii*, discrimination would not be possible with the current method. When determination is not possible with the SOCCET method, samples still need to be sequenced, but the remaining species (*An. aquasalis* and *An. ininii*) were only found sporadically.

**FIGURE 3:**
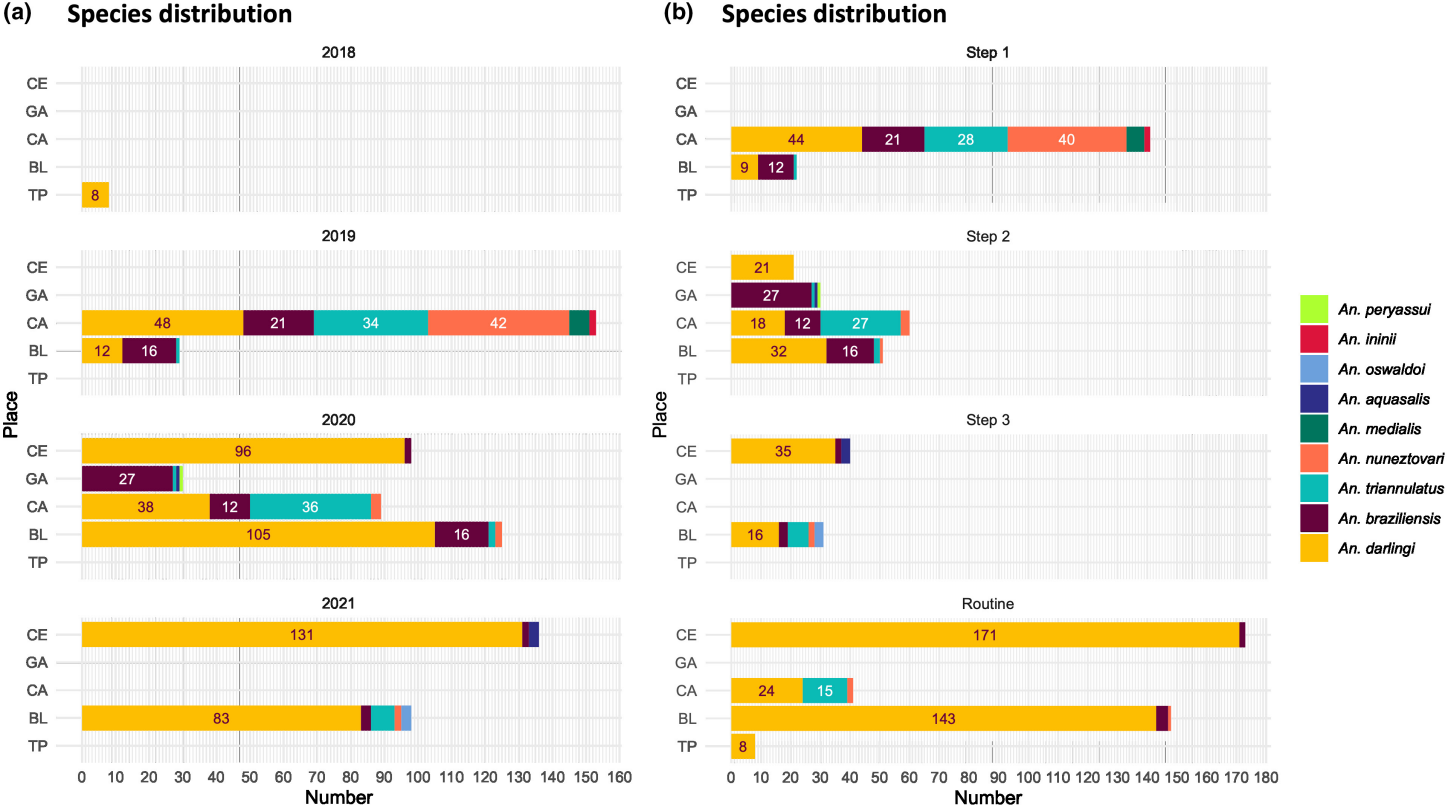
Distribution of *Anopheles* species collected in the five localities between 2018 and 2021, grouped by year (a) or by step (b).

Among our samples, *An. braziliensis*, *An. darlingi*, *An. nuneztovari* and *An. triannulatus* represented 90% of the samples (Figure 3), including 44% of *An. darlingi* after all three development steps and 68% in total, including routine samples. In comparison, we collected only 1, 2, 3, 4 and 6 samples of *An. peryassui*, *An. ininii*, *An. oswaldoi*, *An. aquasalis* and *An. medialis*, respectively. Considering local populations, we collected a mixed population in Cacao, where all four main species were represented, while *An. darlingi* represented a large majority of La Césarée samples (Figure 3a). In Blondin, we also observed a majority of *An. darlingi* in samples, while the proportion of secondary species was variable in both collection years. As depicted in Figure 3b, a larger proportion of *An. darlingi* was identified during routine checks. This is partly linked to our effort to increase sample variability during method development steps by increasing the proportion of samples with a black segment in the hind tarsus and of samples missing colour information.

For each species, we aligned our consensus sequences together with the previous French Guianese sequence published by Vezenegho et al. (2022). For the four main species, we had consensus sequences from different locations. Among *An. braziliensis*, *An. darlingi* and *An. triannulatus*, we did not detect any intraspecific polymorphisms. For *An. nuneztovari*, polymorphisms were not correlated with locations. Interestingly, however, we observed substantial differences between our sequence of *An. oswaldoi* and that of Vezenegho et al.'s, with a 5 ntd difference in amplicon length and 6% dissimilarities in the rest of the sequence. *Anopheles oswaldoi* is known to belong to a species complex encompassing five species and/or subspecies, namely *An. oswaldoi s.s*., *An. oswaldoi* A, *An. oswaldoi* B, *Anopheles konderi*, *Anopheles tadei* (also called *Anopheles* sp. *nr. konderi*) (Saraiva et al., 2018; Saraiva & Scarpassa, 2021). When blasting our sequences and the sequence of Vezenegho et al., it appears our samples, collected in Blondin, cluster with sequences of *An. oswaldoi* B, while Vezenegho's specimen, collected 100 km away at Les Nouragues station, clusters with *An. oswaldoi* A sequences.

**FIGURE 4:**
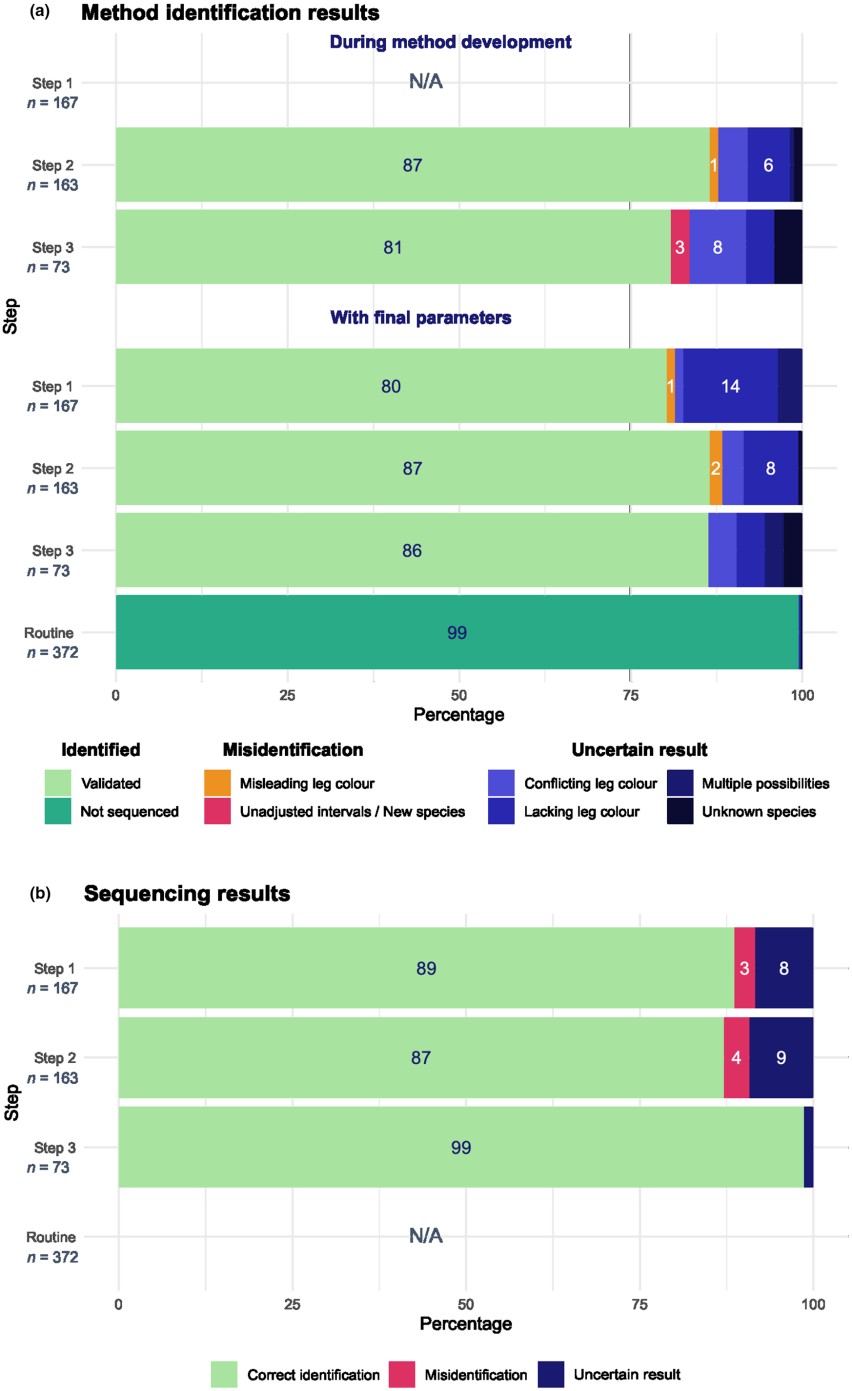
Proportion of samples that were correctly identified, misidentified or not identified using SOCCET (a) and sequencing (b). The SOCCET method was evaluated during development with evolving parameters at each step and after development with the final intervals.

We evaluated the SOCCET method during development, when intervals were evolving at each step (Figure 4a). As step 1 is the primary setting of parameters, the method is not used for identification at this point. At steps 2 and 3, the method allowed us to correctly identify more than 80% of samples. During these steps of development, 1.2 and 2.7% of samples were assigned to the wrong species by the method, respectively, and the errors were detected after validation by sequencing. The misidentifications were due to wrong fifth hind tarsus colour data (2/163) at step 2 and to the need for adjustment of *An. aquasalis* interval (2/73) at step 3. At these steps, 2 and 3, we were not able to determine the species for 12 and 16% of samples respectively (referred to as ‘Uncertain Result’ in Figure 4a), due to four factors: leg colour conflicting with method result, missing leg colour information while amplicon size was at a multiple‐species overlap, interval overlaps and colour corresponding to several species; ITS2 size outside of known intervals, leading to unknown species result.

We also evaluated the SOCCET method by assessing the results of samples from each step with final parameters, for a more accurate evaluation of the final method (Figure 4a). This permitted evaluation of step 1, as well as seeing the changes in identification errors and uncertainties for steps 2 and 3 with the fixed final intervals compared to the changing intervals of method development. 80% of step 1 samples would have been identified correctly by the final method and 1.2% would have been misidentified due to wrong fifth hind tarsus colour data (2/167). We would have been unable to determine the species of 19% of step 1 samples, largely because of missing leg colour metadata. The percentage of correct identifications remained at 80% for step 2 samples with the final parameters and increased to 86% for step 3 samples. With final parameters, misidentifications increased for step 2 samples to 1.8% (3/163; +1) because of wrong leg colours, but decreased for step 3 samples to 0 due to adjustment of intervals. Overall, the proportions of undetermined samples decreased with final parameters.

In routine, over 99% of samples were identified without issues and the remaining uncertainties were very low (0.54%; 2/372) due to rare missing fifth hind tarsus colour and interval overlaps. However, with 93% of *An. darlingi*, mosquito species diversity was lower in routine than in development steps. This is partly due to our effort to increase sample variability during method development, hence the ability of SOCCET to identify species is between what we observed during development and in routine, around 91% if we calculate it over all the samples of all steps.

As we used sequencing as a classical way of species identification, we also looked at the reliability of sequencing in our set‐up after detecting a few inconsistencies between our method and sequencing. After a second sequencing of the corresponding samples, we could quantify that 3.0 and 3.7% of samples at steps 1 and 2, respectively, were misidentified during sequencing (Figure 4b). In addition, 8.4%, 9.2% and 1.4% of samples led to different species identification when sequenced from forward and reverse primers at steps 1, 2 and 3 respectively, which led to uncertain results (Figure 4b). This may be due to cross‐contamination occurring during processing, shipping and/or sequencing.

## 4 DISCUSSION

In this paper, we describe SOCCET, a method of mosquito species identification based on a traditional endpoint PCR and a precise amplicon size detection by capillary electrophoresis, avoiding the need for further treatment of the samples and reducing the need for sequencing by 10‐fold. We found that ITS2 sequence size differences between routinely collected Anopheline species are detectable by capillary electrophoresis. This enabled us to assign species‐specific profiles for nine *Anopheles* species (seven species from the *Nyssorhynchus* subgenus and two from the *Anopheles* subgenus) present in five localities of French Guiana. Identification is based on ITS2 amplicon size information combined with a simple morphological observation, the colour of the fifth hind tarsus. This additional piece of data is quick and easy to acquire, either by eye or under a stereomicroscope, when collecting mosquitoes in the field and does not require any advanced knowledge in taxonomy.

The SOCCET method has the advantage of being simple and relatively quick to set up and can be used routinely in a laboratory, avoiding the need for sequencing for over 90% of samples. During the three steps of method development, we were not able to sequence our samples on‐campus and we had to send samples in 96‐well plates between French Guiana and France for sequencing. For 1.4%–9.2% of samples, it was not possible to obtain definitive results as forward and reverse sequencing outcomes were different. Moreover, we observed that 3.0%–3.7% of sample sequencing results were erroneous in steps 1 and 2. In these cases, forward and reverse sequences were matching with each other but the identification was contradictory with other parameters expected for the species, either with observed leg colour or with measured ITS2 size. Without the implementation of the method, these errors would have gone unnoticed because these parameters would not have been recorded. In case of doubt after the first sequencing, we amplified the ITS2 region again, sent the samples for re‐sequencing and corrected the initial identification when erroneous. Hence, sequencing can be a source of error, especially when samples need to be shipped, as cross‐contaminations may occur during plate processing, shipment and sequencing. In our case, plates were sealed and packaged carefully but we noticed that plate caps that have undergone temperature changes during PCR and storage in the freezer are slightly easier to open. Moreover, we used adhesive PCR plate seals for one shipment of two plates (one from step 1 and one from step 2), which resulted in more frequent misidentifications and uncertain results. During subsequent shipments (steps 2 and 3), we used plate caps that were replaced with new ones just before shipping and observed a relatively low error rate, though we did not investigate this further.

When we evaluate the method with its final interval parameters, missing or erroneous leg colour appears to be the major source of the problem. It rarely led to incorrect identification (1.2% and 1.8% of samples from steps 1 and 2, respectively), but more often led to uncertainties (15%, 11%, 8.2% and 0.27% of samples from steps 1, 2, 3 and routine, respectively). Wrong leg colour specifically led to 1.2%–4.1% inability to determine species, indicating that efforts to ensure correct annotation of this information at the time of capture can significantly improve determination rates. A simple double check by a colleague may be sufficient, as these data are usually collected in a repetitive way, sometimes at night, in a non‐usual environment. Nonetheless, this problem cannot be completely solved, as a small proportion of mosquitoes lack both hind legs. Missing metadata about leg colour concerned 5%–32% of samples. One could decide to exclude samples with missing information from the study, yet we were able to identify the species of 35%–94% of mosquitoes with missing fifth hind tarsus colour information. Across all steps and routines, SOCCET enabled the species determination for 63% of samples without colour information. The fifth hind tarsus colour element is therefore a piece of data that should not be neglected, though if it is missing, identification remains possible in many cases.

Another key point for the reliability of our method is, obviously, the proper use of the capillary electrophoresis device and its components. The channels of the capillary electrophoresis cartridge are prone to clogging if they are used incorrectly, which may generate erroneous results. The only recommendation on this point is to follow the supplier's instructions carefully, check results individually and detect any aberrant results. In case of aberrant results, we either excluded the problematic channels or replaced the cartridge.

Although it was not yet relevant in our routine analyses, we retrospectively advise adding some quality controls alongside routine identifications. One option would be to sequence up to 5% of identified samples of each collection batch or every year, to ensure that no unexpected problem has occurred like a new species appearing in the sampling sites or the need for interval adjustment, even after setting up the method. These re‐sequenced samples may be selected either because their leg colour information is missing or because their ITS2 length is close to interval limits or within overlaps. Particular attention may be given to *An. braziliensis* samples, as *An. marajoara* has a very similar amplicon length and its hind tarsus is also white.

Among the 22 *Anopheles* species known to occur in French Guiana, only a handful are frequently collected with Mosquito Magnets in our routine sampling sites. *Anopheles darlingi* is the most frequent and abundant species in French Guiana and *An. braziliensis*, *An. nuneztovari* and *An. triannulatus* are regularly sampled in different sites across the territory (Dusfour et al., 2013; Roux et al., 2013; Vezenegho et al., 2014). Some species like *An. aquasalis* and *An. peryassui* are restricted to the coastal plain (Adde et al., 2016; Briolant et al., 2020; Dusfour et al., 2013; Vezenegho et al., 2015), while other species like *An. eiseni* and *An. marajoara* were only sampled in strictly forested areas (Dusfour, Jarjaval, et al., 2012; Pommier de Santi et al., 2016). Thus, species with similar ITS2 region size may not all be found in the same localities. Considering such ecological parameters may further help species identification, notably between *An. peryassui* and *An. eiseni* which have similar ITS2 lengths.

While the current method has been developed on *Anopheles* species from French Guiana, this approach may be extended to species identification of individual fieldwork specimens from other regions of the world and in other taxa. Beyond *Anopheles* (*Nyssorhynchus*) genus, our method clearly differentiated our *Anopheles* samples from four outlier mosquitoes of the *Culex* and *Psorophora* genera during step 1, and development steps may require fewer specimens if the species of interest are more distant from each other. More testing would be needed to determine whether all mosquito genera have significantly different profiles from one another and whether, within these genera, species identification is possible. Further, the fifth hind tarsus colour metadata is relevant in the context of our study on *Anopheles* species, but other morphological characteristics could be found for species discrimination among mosquitoes of other genera or subgenera. In the spirit of this method, the selected taxonomic information should be easy to retrieve for non‐specialists. For instance, colouration patterns of the dorsal and lateral parts of the thorax could be relevant for *Aedes* and *Culex* mosquitoes, respectively. Our tool may then facilitate the identification of a broad range of mosquitoes and be particularly advantageous during outbreaks in order to target species that are potential vectors of arboviruses or parasites. In this case, it may require pooling legs from different mosquitoes prior to DNA extraction and analysis, to screen many samples at low cost before checking for the presence of a species of interest in the positive pools. It could be compatible with sampling by non‐experts combined with photographs during collaborative work or citizen science projects.

To compare the interest of our method with currently available methods, several aspects can be considered: the possibility to go back and check results, the level of local diversity and the cost‐effectiveness. Our method allows results to be checked easily as DNA extracts can be stored long term. They can be used for a second similar analysis by PCR and capillary electrophoresis as well as for sequencing of ITS2 or any other sequence, which may notably apply to population genetics or microbiome studies. The other methods of molecular biology have similar advantages, while morphological observations and audio recordings may not unless samples have been properly stored.

In terms of diversity, we detected nine different *Anopheles* species, including four dominant ones. With a higher local diversity, too many overlaps between intervals would reduce the efficiency of the method with the current experimental conditions. The latter can be improved, for instance, by running capillary electrophoresis straight after PCR, which would reduce interval width, facilitating analysis of samples from areas with slightly higher diversity. Sequencing would remain the best option in case of high species diversity. RFLP technique would also be a suitable option in case of medium to high diversity, although it requires additional treatment of the samples after PCR, that is, digestion and gel manipulation, while running capillary electrophoresis just requires opening the wells of the PCR plate after amplification and loading the PCR plate into the apparatus. With a lower diversity of only two or three species, multiplex PCR with species‐specific primers would remain the simplest and cheapest method. However, setting up a multiplex PCR can be complex with a large number of species, as all primers need to have compatible melting temperatures and to be sufficiently different to discriminate species. Hence, our species identification method is an easy, reliable alternative for locally moderate species diversity. Our method is therefore well suited to researchers collecting regularly on the same sites, where they have some knowledge on the local diversity.

Considering costs, capillary electrophoresis requires an initial equipment investment of 20–40 k€. Yet, the processing cost of each sample is lower than 1 € with the proposed method compared to more than 5 € with sequencing (in addition to DNA extraction and PCR, required for both methods). Thus, assessing the initial cost of the device and of capillary electrophoresis reagents on one hand, shipping and sequencing cost of samples on the other hand (and ignoring the cost of labour and our interest in the other applications of the device), our investment in the capillary electrophoresis apparatus would be recovered after 4000 to 5000 samples. Beyond that, more than 5 € per sample can be saved. Hence, episodic needs may rather be addressed with sequencing, while our approach seems reasonable for a laboratory requiring frequent species identification. Alternatively, even with very low consumable cost per sample (Croxatto et al., 2012), the MALDI‐TOF approach requires a 10‐fold higher investment and maintenance budget than capillary electrophoresis. It would therefore require a much higher number of samples (of the order of 100,000) to be more cost‐effective than our method. However, regardless of the method, it is still important to consider that each piece of equipment has multiple functions: in our facility, most gel electrophoresis runs were replaced by capillary electrophoresis.

Our study also allowed us to provide more information on the *Anopheles* species found at five locations in French Guiana. We provide sequencing data that corroborate previous data based on morphological observations, indicating that La Césarée harbours a large majority of *An. darlingi* (Vezenegho et al., 2015). Cacao had a mixed population, mainly encompassing *An. braziliensis*, *An. darlingi*, *An. nuneztovari* and *An. triannulatus*. These species had already been found in Cacao, but then with a larger proportion of *An. triannulatus* and a poor representation of *An. braziliensis* (Roux et al., 2013). We found that Blondin had a variable proportion of mosquito species depending on the year sampled, in line with previous data showing variation between sampling months within a year, and consistent with reported seasonal variations in species distribution (Adde et al., 2017). Our data consolidate previous knowledge on *Anopheles* distribution in French Guiana, which may be helpful to prioritise sampling sites for future studies, whether focusing on a species of interest or on locations with high species diversity. Regarding variations in ITS2 sequences, most of our consensus sequences perfectly or almost perfectly matched previous French Guianese sequences of the same species (Chabanol et al., 2023; Vezenegho et al., 2014). Intraspecies variations were rare, mostly observed between *An. nuneztovari* samples that were not correlated to locations, corroborating previously known variability in ITS2 sequences of this species (Onyabe & Conn, 1999) and the observation of multiple peaks in most samples. However, our samples of *An. oswaldoi*, collected in Blondin, apparently belong to *An. oswaldoi* B, which is closer to *An. oswaldoi s.s*., while Vezenegho et al.'s sample collected in Les Nouragues station, a pristine rainforest area, clusters with *An. oswaldoi* A, closer to *An. konderi*. This result is in line with previous observations indicating that *An. oswaldoi* A and B can be found at similarly distant sites in the Amapa, the Brazilian state bordering French Guiana (Saraiva et al., 2018). Whether these phylogenetic groups are subspecies or species is as yet unclear, as species boundaries of the *An. oswaldoi‐konderi* complex are still under characterisation (Saraiva & Scarpassa, 2021).

In summary, we introduce PCR and capillary electrophoresis combined with a simple morphological observation as an alternative and convenient method to discriminate between species of field‐collected samples. We specifically deployed this method to identify *Anopheles* species, mainly from the *Nyssorhynchus* subgenus, found in five localities with moderate diversity representing different landscapes in French Guiana. This method allows us to reduce the proportion of samples requiring sequencing by 90%, thereby saving both time and money.

## AUTHOR CONTRIBUTIONS

**Estelle Chabanol:** Conceptualization (lead); data curation (lead); formal analysis (lead); funding acquisition (equal); investigation (lead); methodology (lead); project administration (lead); validation (lead); visualization (lead); writing – original draft (lead); writing – review and editing (lead). **Ottavia Romoli:** Investigation (supporting); writing – review and editing (supporting). **Stanislas Talaga:** Investigation (supporting); writing – review and editing (supporting). **Yanouk Epelboin:** Investigation (supporting); writing – review and editing (supporting). **Katy Heu:** Investigation (supporting). **Ghislaine Prévot:** Conceptualization (supporting); funding acquisition (supporting); project administration (supporting); supervision (supporting); writing – review and editing (supporting). **Mathilde Gendrin:** Conceptualization (lead); formal analysis (lead); funding acquisition (lead); methodology (lead); project administration (lead); supervision (lead); validation (lead); visualization (lead); writing – original draft (lead); writing – review and editing (lead).

## FUNDING INFORMATION

This research was funded by French Government's Agence Nationale de la Recherche for Laboratoires d'Excellence for Integrative Biology of Emerging Infectious Diseases (ANR‐10‐LABX‐0062‐IBEID) to M.G., Agence Nationale de la Recherche for Jeunes Chercheuses Jeunes Chercheurs for Mosquito Microbiota (ANR‐18‐CE15‐0007‐MosMi) to M.G., Agence Nationale de la Recherche for Laboratoires d'Excellence (ANR‐10‐LABX‐2501) to G.P., Ecole Doctorale 587 Université de Guyane 3‐year PhD Studentship to E.C. and a Fondation pour la Recherche Médicale fourth year PhD Studentship (FDT202204015140) to E.C.

## CONFLICT OF INTEREST STATEMENT

The authors declare no conflict of interest.

### OPEN RESEARCH BADGES

This article has earned an Open Data badge for making publicly available the digitally‐shareable data necessary to reproduce the reported results. The consensus sequences are available in GenBank (NCBI, NIH) database with accession numbers from OR149213 to OR149254: https://www.ncbi.nlm.nih.gov/nuccore/?term=OR149213:OR149254[accn].

## Supporting information


Appendix S1

Data S1

Data S2

Data S3

Data S4

Data S5

## Data Availability

42 consensus sequences are accessible in the GenBank (NCBI, NIH) database with accession numbers from OR149213 to OR149254 (Chabanol et al., 2023). All forward and reverse sequences are provided in Data S1, and consensus sequence alignment can be found in Data S2 file. Method development steps and routine analysis are available in Data S3. R data and script for plots are available in Data S4 and S5.
