# Supplementary material for: Using capillary electrophoresis to identify Anopheline species in routine sampling sites
Source: Ecol Evol. 2024 Mar 12;14(3):e10782. doi: 10.1002/ece3.10782 (PMC10933085; doi:10.1002/ece3.10782)
Supplement: Supplementary file 1 — Appendix S1 [file ECE3-14-e10782-s004.docx]

**Appendix**

**Figure S1:** Species-specific ITS2 amplicon sizes are visible on an agarose gel **(A)** but more precisely measured with capillary electrophoresis including internal reference size controls **(B)**. Additionally, capillary electrophoresis allows quality check of fragment amplification and migration **(C)**.


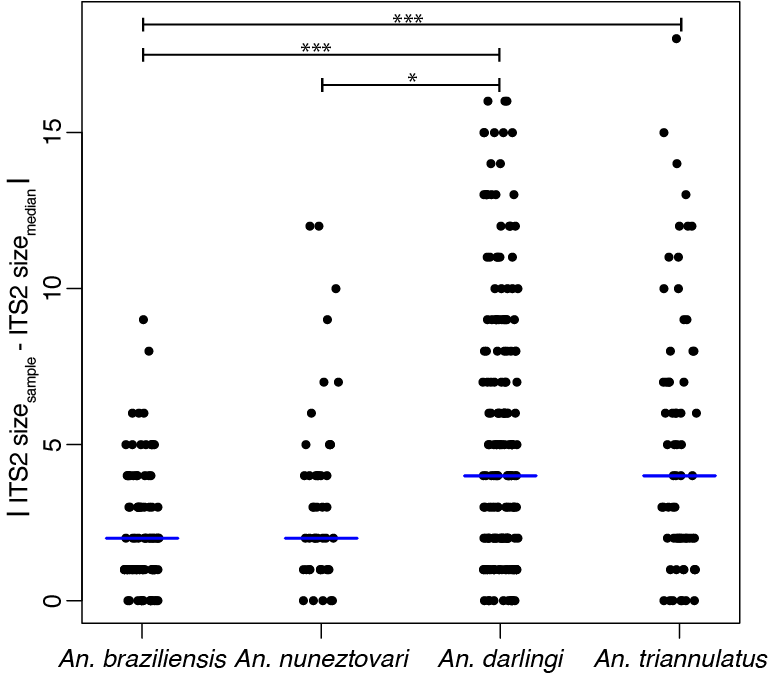


**Figure S2:** Absolute values of the deviation from the median of measured ITS2 length in each sample. Data are shown for the four species where a high number of samples was collected. Each dot represents an individual observation and blue bars represent medians. Data were analysed via a Dunn (1964) Kruskal-Wallis multiple comparison (p-adjustment method: Holm) and non-significant differences are not indicated.

| ***Anopheles*** | **ITS2 Amplicon Length** | **n** | **Accession Number (GenBank)** | **Reference (PMID)** |
| --- | --- | --- | --- | --- |
| ***eiseni*** | 400 | 1 | KP994161.1 | Unpublished Vezenegho,S.B., Dusfour,I., Girod,R. and Briolant,S. |
|  | 467 | 1 | AF462380.1 | 15958027 Marrelli et al. 2005 |
| ***medialis*** | 413 | 1 | KP994163.1 | Unpublished Vezenegho,S.B., Dusfour,I., Girod,R. and Briolant,S. |
| ***costai*** | 421 | 1 | KP994167.1 | Unpublished Vezenegho,S.B., Dusfour,I., Girod,R. and Briolant,S. |
| ***nimbus*** | 442 | 1 | KP994159.1 | Unpublished Vezenegho,S.B., Dusfour,I., Girod,R. and Briolant,S. |
| ***peryassui*** | 466 | 1 | KF698915.1 | 25774795 Gomez et al. 2015 |
|  | 467 | 5 | KF698916.1 KF698914.1 KF698913.1 KF698912.1 KF698911.1 |  |
|  | 468 | 1 | AF461755.1 | 15958027 Marrelli et al. 2005 |
| ***neivai*** | 477 | 1 | KP994164.1 | Unpublished Vezenegho,S.B., Dusfour,I., Girod,R. and Briolant,S. |
| ***oswaldoi*** | 482 | 7 | AY679155.1 AY679154.1 AY679153.1 AY679152.1 AY679151.1 AY679150.1 AY679149.1 | Unpublished Ruiz,F., Quinones,M., Calle,D. and Linton,Y.-M. |
|  | 484 | 2 | AF056318.1 AF055070.1 | 10593066 Marrelli et al. 1999 |
|  | 485 | 1 | AF055072.1 |  |
|  | 487 | 1 | AF055069.1 |  |
|  | 488 | 1 | AF056317.1 |  |
|  | 489 | 1 | AF055068.1 |  |
|  | 492 | 1 | AF055071.1 |  |
|  | 488 | 1 | KP994153.1 | Unpublished Vezenegho,S.B., Dusfour,I., Girod,R. and Briolant,S. |
|  | 489 | 5 | EU636809.1 EU636808.1 EU636807.1 EU636806.1 EU636803.1 | 19058619 Sallum et al. 2008 |
|  | 491 | 3 | EU636805.1 EU636804.1 EU636802.1 |  |
| ***aquasalis*** | 483 | 1 | AF462376.1 | 15958027 Marrelli et al. 2005 |
|  | 485 | 1 | KP994162.1 | Unpublished Vezenegho,S.B., Dusfour,I., Girod,R. and Briolant,S. |
| ***marajoara*** | 487 | 1 | KP994160.1 | Unpublished  Vezenegho,S.B., Dusfour,I., Girod,R. and Briolant,S. |
|  | 487 | 1 | AY028127.1 | Unpublished Linton,Y.M., Sierra,D.M., Quinones,M.L. and Harbach,R.E. |
| ***braziliensis*** | 487 | 1 | AF461753.1 | 15958027 Marrelli et al. 2005 |
|  | 488 | 1 | KP994156.1 | Unpublished Vezenegho,S.B., Dusfour,I., Girod,R. and Briolant,S. |
| ***ininii*** | 495 | 1 | KP994152.1 | Unpublished Vezenegho,S.B., Dusfour,I., Girod,R. and Briolant,S. |
| ***nuneztovari*** | 498 | 1 | KP994154.1 | Unpublished Vezenegho,S.B., Dusfour,I., Girod,R. and Briolant,S. |
|  | 500 | 1 | AF461749.1 | 15958027 Marrelli et al. 2005 |
|  | 500 | 38 | AY028128.1 AY028126.1 - AY028081.1 | Unpublished Linton,Y.M., Sierra,D.M., Quinones,M.L. and Harbach,R.E. |
|  | 500 | 2 | GU477282.1 GU477281.1 | Unpublished Cienfuegos,A.V. and Correa,M.M. |
| ***triannulatus*** | 534 | 1 | JX852289.1 | 22949519 Rosero et al. 2012 |
|  | 536 | 16 | JX852318.1 JX852317.1 JX852314.1 - JX852311.1 JX852300.1 - JX852296.1 JX852294.1 - JX852290.1 |  |
|  | 553 | 2 | JX852284.1 JX852283.1 |  |
|  | 560 | 4 | JX852319.1 JX852315.1 JX852310.1 JX852295.1 |  |
|  | 561 | 1 | JX852316.1 |  |
|  | 565 | 14 | JX852320.1 JX852309.1 - JX852301.1 JX852288.1 - JX852285.1 |  |
|  | 536 | 1 | AF462377.1 | 15958027 Marrelli et al. 2005 |
|  | 564 | 1 | KP994151.1 | Unpublished Vezenegho,S.B., Dusfour,I., Girod,R. and Briolant,S. |
|  | 565 | 2 | GU477280.1 GU477279.1 | Unpublished Cienfuegos,A.V. and Correa,M.M. |
| ***darlingi*** | 543 | 2 | GU477278.1 GU477276.1 | Unpublished Cienfuegos,A.V. and Correa,M.M. |
|  | 544 | 1 | GU477277.1 |  |
|  | 545 | 1 | AF462389.1 | 15958027 Marrelli et al. 2005 |
|  | 546 | 1 | AF462388.1 |  |
|  | 546 | 1 | KP994157.1 | Unpublished Vezenegho,S.B., Dusfour,I., Girod,R. and Briolant,S. |
| ***minor*** | 680 | 1 | KP994158.1 | Unpublished Vezenegho,S.B., Dusfour,I., Girod,R. and Briolant,S. |

**Table S1:** References of ITS2 fragment sizes of 15 *Anopheles* species found in French Guiana. Amplicon sequences were found in GenBank (NCBI, NIH) database and lengths were adjusted (trimmed or extended) to correspond to sequence sizes between our forward (5’-TGTGAACTGCAGGACACAT-3’) and reverse (5’-TATGCTTAAATTCAGGGGGTAG-3’) primers (included).

| ***Anopheles*** | **Sequence** | **Step 1 (Initial)** | **Step 2 (Adjusted)** | **Step 3 (Final)** | **Identified** | **Uncertain** | **Misid.** |
| --- | --- | --- | --- | --- | --- | --- | --- |
| ***triannulatus*** | 564 bp | 566-595 bp | 566-595 bp | 563-595 bp | 59/66 (89%) | 7/66 (11%) | 0/66 (0%) |
| ***darlingi*** | 546 bp | 548-575 bp | 548-576 bp | 548-576 bp | 146/176 (83%) | 25/176 (14%) | 5/176 (2.8%) |
| ***nuneztovari*** | 498-501 bp | 493-509 bp, peaks ~500 | 493-509 bp, peaks ~500 | 493-509 bp, peaks ~500 | 41/46 (89%) | 5/46 (11%) | 0/46 (0%) |
| ***ininii*** | 496 bp | 486-494 bp | 486-494 bp | 486-494 bp | 0/2 (0%) | 2/2 (100%) | 0/2 (0%) |
| ***aquasalis*** | 485 bp |  | 484-484 bp | 483-490 bp | 2/4 (50%) | 2/4 (50%) | 0/4 (0%) |
| ***braziliensis*** | 488 bp | 479-493 bp | 479-493 bp | 479-493 bp | 78/94 (83%) | 16/94 (17%) | 0/94 (0%) |
| ***oswaldoi*** | 484 bp |  |  | 475-482 bp | 2/3 (67%) | 1/3 (33%) | 0/3 (0%) |
| ***peryassui*** | 467 bp |  | 467-467 bp | 467-467 bp | 1/1 (100%) | 0/1 (0%) | 0/1 (0%) |
| ***medialis*** | 413 bp | 415-422 bp | 415-422 bp | 415-422 bp | 5/6 (83%) | 1/6 (17%) | 0/6 (0%) |

**Table S2:** ITS2 size intervals and modifications of the intervals at development steps 1, 2 and 3 compared to the observed sequence lengths for each *Anopheles* species. Species-specific identification results using the final intervals are also indicated. Identified – correct identification; Uncertain – colour + capillary electrophoresis method does not allow to conclude; Misid. (Misidentification) – SOCCET leads to an erroneous identification.

|  | | **Amplicon Size** | | | | |
| --- | --- | --- | --- | --- | --- | --- |
| **species** | **sample** | **Initial Result** | **PCR1 DAY1** | **PCR1 DAY2** | **PCR2 DAY1** | **PCR2 DAY2** |
| ***An. darlingi*** | **CA15** | 548 | 562 | 564 | 559 | 561 |
|  |  |  | 562 | 562 | 558 | 560 |
|  |  |  | 561 | 562 | 558 | 556 |
|  | **nCE65** | 559 | 566 | 564 | 564 | 561 |
|  |  |  | 566 | 563 | 564 | 561 |
|  |  |  | 568 | 571 | 563 | 569 |
|  | **CE158** | 573 | 562 | 569 | 560 | 564 |
|  |  |  | 562 | 565 | 559 | 563 |
|  |  |  | 562 | 565 | 559 | 561 |
|  | **nCE44** | 574 | 561 | 563 | 558 | 559 |
|  |  |  | 560 | 561 | 557 | 558 |
|  |  |  | 564 | 570 | 560 | 566 |
| ***An. triannulatus*** | **nBL89** | 563 | 577 | 577 | 576 | 577 |
|  |  |  | 577 | 577 | 574 | 572 |
|  |  |  | 581 | 586 | 579 | 583 |
|  | **CA76** | 573 | 577 | 579 | 573 | 575 |
|  |  |  | 575 | 578 | 573 | 575 |
|  |  |  | 576 | 580 | 575 | 575 |
|  | **nBL14** | 574 | 574 | 578 | 574 | 576 |
|  |  |  | 575 | 578 | 573 | 575 |
|  |  |  | 576 | 585 | 578 | 583 |
|  | **CA85** | 588 | 575 | 582 | 573 | 574 |
|  |  |  | 575 | 581 | 572 | 574 |
|  |  |  | 577 | 584 | 575 | 579 |

**Table S3:** *Anopheles darlingi* and *An. triannulatus* DNA samples from both extremes of their respective intervals during steps 1 – 3 (“expected length”) were re-amplified in two separate PCR and capillary electrophoresis migration was performed twice for each PCR product on two different days. There is no significant effect of the expected length on the averaged amplicon size between PCRs (Kruskal-Wallis rank sum test; *An. darlingi* – p = 0.16, chi-squared = 5.2, df = 3; *An. triannulatus* – p = 0.93, chi-squared = 0.44, df = 3).
